# Antibacterial and Antioxidant Activity of Extracts from Rose Fruits (*Rosa rugosa*)

**DOI:** 10.3390/molecules25061365

**Published:** 2020-03-17

**Authors:** Andrzej Cendrowski, Karolina Kraśniewska, Jarosław L. Przybył, Agnieszka Zielińska, Stanisław Kalisz

**Affiliations:** 1 Department of Food Technology and Assessment, Institute of Food Sciences, Warsaw University of Life Sciences-SGGW, Nowoursynowska 159C Str., 02-776 Warsaw, Poland; stanislaw_kalisz@sggw.pl; 2 Department of Food Biotechnology and Microbiology, Institute of Food Sciences, Warsaw University of Life Sciences-SGGW, Nowoursynowska 159C Str., 02-776 Warsaw, Poland; 3 Department of Vegetable and Medicinal Plants, Institute of Horticulture Sciences, Warsaw University of Life Sciences-SGGW, Nowoursynowska 159 Str., 02-776 Warsaw, Poland; jaroslaw_przybyl@sggw.pl; 4 Chair of Physical Pharmacy and Bioanalysis, Department of Physical Chemistry, Faculty of Pharmacy, Medical University of Warsaw, Banacha 1 Str., 02-097 Warsaw, Poland; agnieszka.zielinska@wum.edu.pl

**Keywords:** plant material, freeze-dried extract, minimum inhibitory concentration (MIC), minimum bactericidal concentration (MBC), time-kill method

## Abstract

The aim of the present study was to determine the antioxidant and antimicrobial properties in freeze-dried extracts of rose fruits (*Rosa rugosa*) obtained using various extraction techniques and to determine the effect of a selected extract on bacterial survival in model fluids imitating protein food. Ethanolic extracts from rose fruits showed higher antioxidant activity compared to other tested extracts. The rose fruits aqueous extract showed the highest inhibitory activity against most of the 10 bacterial strains tested. From the group of Gram-positive bacteria, the *Bacillus cereus* strain proved to be the most sensitive to the action of the rose extract. From the Gram-negative bacteria: *Escherichia coli* and *Klebsiella pneumoniae* were the most sensitive. The reduction in the number of bacterial cells in matrices imitating protein food depended on the concentration of the aqueous extract used. However, at none of the concentrations used was a complete inhibition of bacterial growth observed. We have confirmed that the traditional extraction and freeze-drying of rose fruits is still suitable for the food industry due to obtaining extracts with good antibacterial and antioxidant properties and the use of bio-solvents, such as water or ethanol, which are easily available in high purity and completely biodegradable.

## 1. Introduction

Owing to the high toxicity of synthetic compounds, the search for new antiradical, as well as antimicrobial substances, still remains a challenge for modern science. Knowing the variation of the antioxidant and antibacterial activity is very important for choosing the plant material that can be used in food production, health industry, and future breeding programs [1]. 

Natural antioxidants added to food affect the reduction of free radicals, the chelation of metal ions that are catalysts in the formation of free radicals, the inhibition of the activity of oxidizing enzymes, reducing the amount of secondary and primary products formed as a result of the oxidation process, inhibiting the formation of formaldehyde, and slowing down the formation of metmyoglobin [2]. 

Plant tissues are naturally rich in nutritive or therapeutically active products of plant secondary metabolism. Many papers have reported that phenolic compounds and polyphenol extracts derived from natural plants can delay age-related decline and extend lifespan across a variety of species [3,4]. The diet rich in polyphenols has been associated with reducing the risk for cardiovascular diseases, cancer, and other diseases. These compounds have antioxidant, anti-inflammatory, and anticarcinogenic properties. According to Pandey and Rizvi [5], polyphenols and flavonoids are antioxidants that provide a significant protection of the human body against some diseases, including cancer, diabetes, neurodegenerative, and cardiovascular problems. Antioxidants can help to neutralize reactive oxygen species generated in human body, reducing tissue damage and alleviating oxidative stress [6]. 

Plant extracts introduced into food favorably affect its organoleptic properties, giving the products a specific taste and smell, and at the same time are a source of natural substances containing bioactive compounds, among others polyphenolic compounds with antioxidant properties. Thanks to the antioxidant properties, plant extracts can affect the extension of shelf life and in many cases improve the microbiological quality of food. Research is increasingly pointing to the possibility of using antioxidant and antimicrobial properties of plant extracts and their introduction into food products as natural preservatives [7,8,9,10,11]. 

Natural plant substances, especially extracts, are very often used by the meat industry. Many authors indicate in their research that antioxidants should be used to protect the meat raw material against the negative process occurring during storage, including the oxidation of proteins and fats [2,10]. An example would be the use of rosemary and sage extracts in meat processing. These extracts slowed down the lipid oxidation process, thus extending the shelf life of meat. The study found that the addition of rosemary extract to boiled or roasted beef improved the product’s organoleptic properties. In turn, in a physicochemical assessment, it was found that the addition of the extract reduced the amount of leakage in the thawing process. In addition, no studies have found any effect of storage on the TBARS value (lipid oxidation index), which indicates that the addition of the extract to the raw material inhibits the lipid oxidation process. It was also observed that the addition of the extract had a positive effect on the color of the product. The extracts used contributed to inhibiting the degradation of the heme pigments responsible for the proper color of meat and meat preparations [7,8]. It has also been proven that rosemary extract not only affects the inhibition of TBARS growth but also results in better protection of the color of pork sausage during freezing and refrigeration storage compared to synthetic antioxidants [7].

The antimicrobial effect of plant extracts is influenced, among others, by the plant species, cultivation method, its chemical composition, as well as the extraction method used and the type of solvent used. The mechanism of action of biologically active compounds of plant origin on a bacterial cell is diverse. These compounds can cause cell wall degradation, cytoplasmic membrane destabilization, the inactivation of the intracellular enzymes responsible for cell metabolic processes, and, in turn, the inhibition of replication and transcription processes through interaction with nucleic acids [12]. 

The polyphenols present in plant extracts, such as flavonoids or tannins, due to the presence of -OH groups, tend to incorporate into microorganism’s membrane and cell wall, which in turn leads to a change in their fluidity and permeability. Polyphenolic compounds can also inhibit the synthesis of DNA and RNA, polysaccharides, enzymes, and proteins. This causes a violation of the enzyme system as well as a weakening of biochemical stability and a weakening of the membrane potential, which, in consequence, contributes to the death of the microbial cell [13,14]. In addition, the compounds contained in plant extracts can affect the protein layer of the cell structure in which ATPase is found, enzymes surrounded by lipid molecules. The first mechanism assumes that the lipophilic hydrocarbon components accumulate in the bilayer protein-lipid structure, affecting the structural and functional properties of these membranes. In the second mechanism, however, lipophilic hydrophobic systems act directly on protein molecules present in the microbial cell [15,16].

The most common and the simplest method of obtaining bioactive substances from plant material is extraction. The extraction of bioactive compounds depends on several factors, such as the extraction technique, raw materials, and the extraction solvent that are used [17]. The techniques can be classified into conventional or non-conventional. Conventional techniques require the use of organic solvents, temperature, and agitation. Modern techniques, or non-conventional techniques, are green or clean techniques due to a reduced use of energy and the implementation of organic solvents, which are beneficial in relation to the environment [18].

Rose fruits deserve special attention as a potential source of natural antioxidants and other bioactive substances. They have been discovered to be rich in polyphenols (including tannins, flavonoids, phenolic acids, and anthocyanins), carotenoids (mainly lycopene, β-cryptoxanthin, β-carotene, rubixanthin, gazaniaxanthin, and zeaxanthin), polysaccharides, essential oil (contains alcohols, aldehydes, monoterpenes, sesquiterpenes, and esters), polyunsaturated fatty acids (in seeds), vitamin A, B_1_, B_2_, B_6_, D, E, and K, and mineral nutrients (mainly phosphorus, potassium, calcium, magnesium, manganese, and zinc) [19,20,21,22,23,24,25]. In Poland, roses from the species *Rosa rugosa* are grown on commercial plantations. The limitation in the technological use of rose fruits is their low durability. The fact that such fruits are harvested during collective maturity means that they must be processed quickly. One of the options for preserving both fruits and the extracts obtained from them may be the use of a freeze-drying process.

Despite the many years of tradition of using rose fruits and the enormous technological potential of this raw material, there is little literature data describing the impact of the method of obtaining extracts by different extraction techniques and their freeze-drying on the preservation of their antioxidant and antimicrobial properties.

The aim of the present study was to determine the antioxidant and antimicrobial properties in freeze-dried extracts of rose fruits (*Rosa rugosa*) obtained using various extraction techniques and determining the effect of a selected extract on bacterial survival in model fluids imitating protein food.

## 2. Results and Discussion

### 2.1. Antibacterial Activity of Extracts

#### 2.1.1. Determination of the Minimum Inhibitory Concentration (MIC) and the Minimum Bactericidal Concentration (MBC) of Rose Fruits Extracts

**Figure 1:**
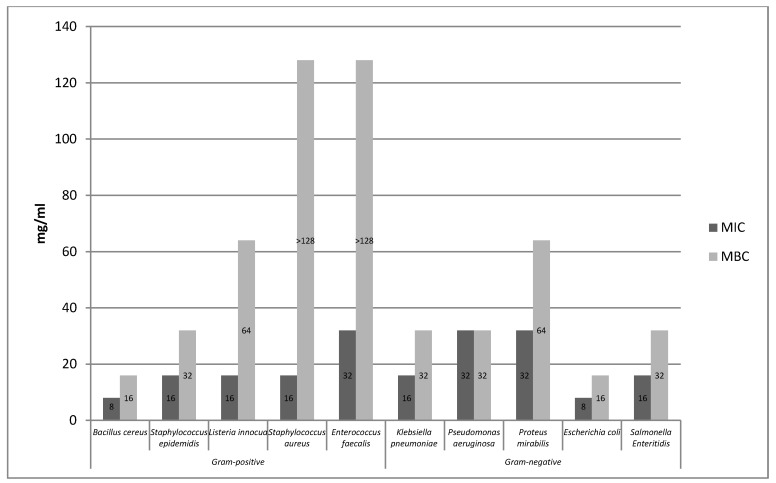
Minimum inhibitory concentration (MIC) and minimum bactericidal concentration (MBC) of aqueous rose fruits extract (A) relative to Gram-positive and Gram-negative bacterial test strains.

**Figure 2:**
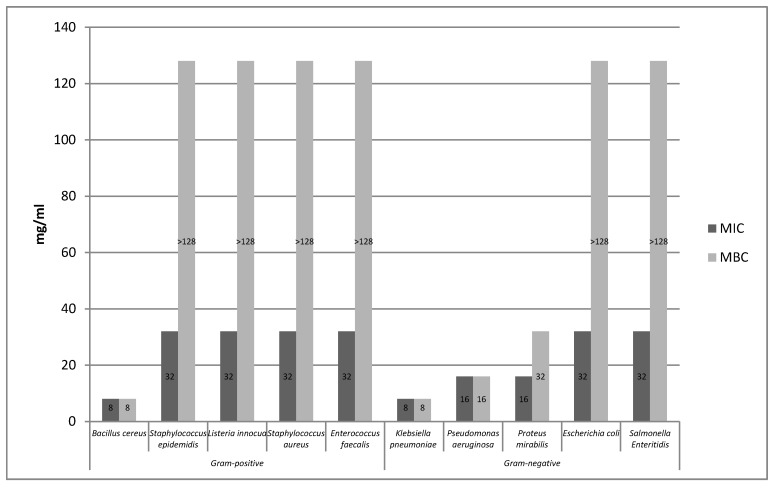
Minimum inhibitory concentration (MIC) and minimum bactericidal concentration (MBC) of ethanolic rose fruits extract (E) relative to Gram-positive and Gram-negative bacterial test strains.

**Figure 3:**
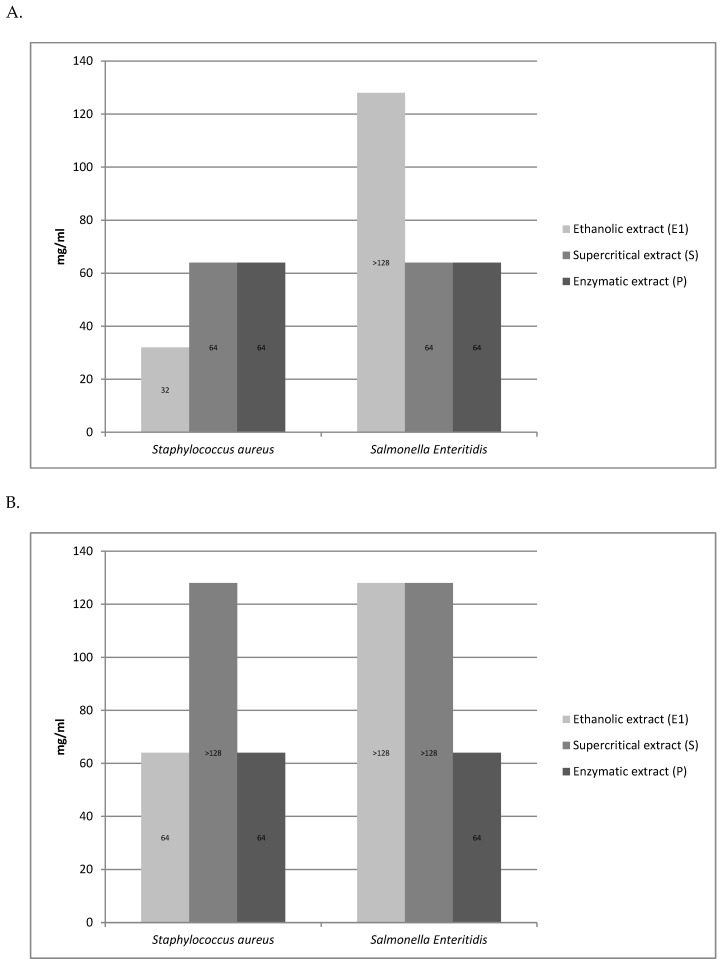
Comparison of the minimum inhibitory concentration (MIC) (**A**) and the minimum bactericidal concentration (MBC) (**B**) of the obtained extracts (E1, S, P) against selected Gram-positive and Gram-negative bacterial test strains.

Tested extracts showed antimicrobial activity against the model bacterial strains (Figure 1, Figure 2 and Figure 3). Based on the conducted research, it was found that the aqueous rose extract strongly inhibited the growth of *Bacillus cereus* bacteria. The most resistant strains among Gram-positive bacteria turned out to be *Staphylococcus aureus* and *Enterococcus faecalis*, while the bactericidal effect of the extract was above the concentration range tested. In the case of Gram-negative bacteria, it was found that aqueous rose extract strongly inhibited the growth of *Escherichia coli. Pseudomonas aeruginosa* and *Proteus mirabilis* turned out to be the most resistant strains of Gram-negative bacteria. 

Based on the conducted research, it was found that the ethanolic extract of rose fruits strongly inhibited the growth of *Bacillus cereus* bacteria (Figure 2). In the case of the remaining Gram-positive bacteria, it was found that the bacteriostatic effect was 32 mg/mL, whereas the bactericidal effect of the extract, which was above the tested concentration range was not established. Of the Gram-negative strains, the highest bacteriostatic effect was observed against *Klebsiella pneumoniae.*

The minimum inhibitory concentration and the minimum bactericidal concentration of the tested rose fruits extracts against the two test strains tested (*S. aureus, Salmonella* Enteritidis) are shown in Figure 3.

The tested extracts (A, E) showed antimicrobial activity against all 10 bacterial strains*. B. cereus*, *E. coli*, and *Klebsiella pneumoniae* were the most sensitive to the extracts. In turn, the most resistant strains were *Enterococcus faecalis* and *Proteus mirabilis*. In the case of the aqueous extract, the highest sensitivity was observed in the case of the Gram-positive *Bacillus cereus* strain, while, among the Gram-negative bacteria, *Escherichia coli* proved to be the most sensitive. *Pseudomonas aeruginosa*, *Proteus mirabilis*, and *Enterococcus faecalis* strains proved to be the most resistant to the action of the aqueous extract. The ethanolic extract (E) showed a strong bacteriostatic effect on the strains of *Bacillus cereus* and *Klebsiella pneumoniae*, while the highest resistance to the said extract was shown by the bacteria *Staphyloccocus aureus*, *Staphylococcus epidermidis*, *Listeria innocua*, *Enterococcus faecalis*, *Salmonella* Enteritidis, and *Proteus mirabilis*. In the case of the ethanol extract (E1), the same bacteriostatic effect on the *S. aureus* strain was observed as for the ethanol extract (E). However, the bacteriostatic and bactericidal activity of the ethanol extract (E1) on the *S.* Enteritidis strain was not established because it was above the concentration range tested. The supercritical (S) and enzymatic extract (P) showed weaker bacteriostatic activity for the two tested strains (*S. aureus*, *S.* Enteritidis) compared to other extracts. The bactericidal activity of the supercritical extract (S) was not demonstrated for the *S. aureus* and *S.* Enteritidis strains because it was above the concentration range tested.

Based on the conducted research, it was found that the aqueous extract (A) showed higher antibacterial activity compared to the ethanolic extract (E). Of the group of 10 bacteria tested, the aqueous extract showed high activity against six strains (i.e., *Bacillus cereus*, *Staphylococcus aureus*, *Staphylococcus epidermidis*, *Listeria innocua*, *Escherichia coli*, and *Salmonella* Enteritidis), and, in turn, the water-ethanolic extract against three strains (i.e., *Bacillus cereus Klebsiella pneumoniae*, *Pseudomonas aeruginosa*). The antimicrobial effect of extracts obtained from *Rosa rugosa* rose fruits is probably due to the presence of phenolic compounds in their chemical composition. Phenolic compounds contained in the extracts lead to changes in the permeability of the cell wall, which in turn leads to ionic disorders. In addition, said compounds may interfere with membrane function and interact with membrane proteins. These actions may damage its structure and functionality. The effectiveness of phenolic compounds on a bacterial cell may depend on their concentration in the extract. At low concentration, these compounds affect the activity of enzymes, while at high concentration they cause the denaturation of proteins present in microbial cells [26]. Literature reports indicate that phenolic compounds are likely to have a toxic effect at a membrane level. In addition, a high degree of correlation was observed between the toxicity and hydrophobicity of various phenolic compounds. Phenol affects the functioning of the membrane by changing the ratio of proteins to lipids in the membrane, causing the leakage of potassium ions [14,27,28]. Flavonoids contained in *Rosa rugosa* rose fruits also damage bacteria and cause the aggregation of bacterial cells, which may result in bacterial cell death [29]. The strong antimicrobial effect of the extracts analyzed in the work may also be associated with the presence of hydrophobic compounds (tocopherol, carotenoids) that cause the disruption of the bacterial cell membrane [26]. In the experiment conducted by Halawani [30], the action of an aqueous and ethanolic extract from *Rosa damascena* was tested against 10 microorganisms. According to the conducted research, the largest bacteriostatic and bactericidal activity was characterized by ethanol extract. *Pseudomonas aeruginosa* and *Escherichia coli* proved to be the most sensitive to the action of the ethanol extract, where MIC and MBC were 62.5 μL*/*mL. In the research conducted by Ulusoy and Gulgun [31], the effects of damask rose extracts against Gram-positive and Gram-negative bacterial strains of *Bacillus subtilis*, *Staphylococcus aureus*, *Pseudomonas aeruginosa*, and *Escherichia coli* were analyzed. It was found that the water–ethanol extract showed bacteriostatic activity in relation to all bacterial strains, whereas no bactericidal activity was noted. Ozkan et al. [32] compared the effect of aqueous and ethanolic extracts obtained from dried and fresh damask rose petals on pathogenic bacteria. Both tested extracts showed antibacterial properties in relation to the majority of tested pathogens. Only *Escherichia coli* did not show any antibacterial activity. *Salmonella* Enteritidis turned out to be the most sensitive. It was also found that the extract of fresh petals was more effective than the extract obtained from dried rose petals. In the analysis carried out by Abu-Shanab et al. [33], the effect of four extracts obtained from various plant species, including Damask rose, was examined. The antimicrobial activity of the extracts was tested by determining the minimum inhibitory concentration (MIC) and the minimum bactericidal concentration (MBC) against the Gram-positive bacterium *Staphylococcus aureus*. The alcoholic extract from damask rose showed higher efficacy against the pathogen compared to other plant extracts of lemon balm, mint, and marshmallow. The minimum inhibitory concentration and the minimum combative concentration of the alcoholic extract were, respectively, from 0.395 to 0.780 mg*/*mL for MIC and 1.563 to 3.125 mg*/*mL for MBC. Larger MIC and MBC values for the studied *Rosa rugosa* rose fruits extracts compared to the results obtained by other authors may result from the different content of individual biological active ingredients found in individual rose cultivars, which significantly affect bacterial growth. Qualitative and quantitative composition of extracts obtained from various rose species and their biological activity may be subject to various types of environmental, genetic, and ontogenetic variability [34].

#### 2.1.2. Survival of Gram-Negative and Gram-Positive Bacteria by the Time-Kill Method in Model Fluids Imitating Protein Food

##### Study of the Survival of *Salmonella* Enteritidis in Model Fluids Imitating Protein Food

**Figure 4:**
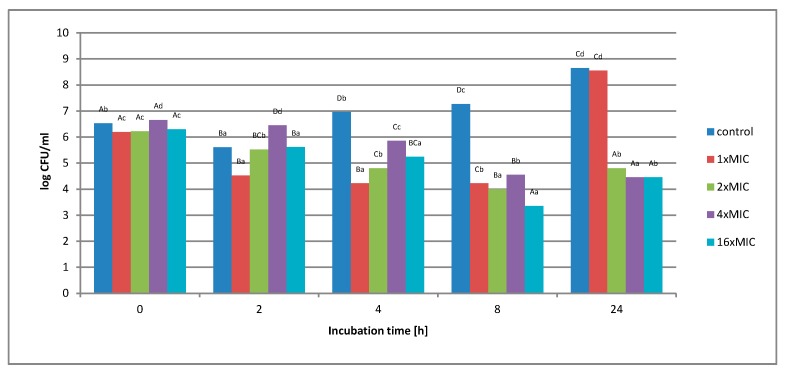
The effect of the aqueous extract at the concentration of 1 × MIC, 2 × MIC, 4 × MIC, and 16 × MIC on the survival of *Salmonella* Enteritidis bacteria in the control matrix during 24 h of incubation. A, B, C, D—the effect of matrix type on the change in the number of bacteria at the same time of storage; a, b, c, d—the effect of incubation time on changing the number of bacteria.

Figure 4 shows the effect of an aqueous extract on the survival of *Salmonella* Enteritidis in a control matrix, i.e., in Tryptic Soy Broth (TSB) medium. Regardless of the concentration of the extract used, no complete inhibition of microbial growth was found.

**Figure 5:**
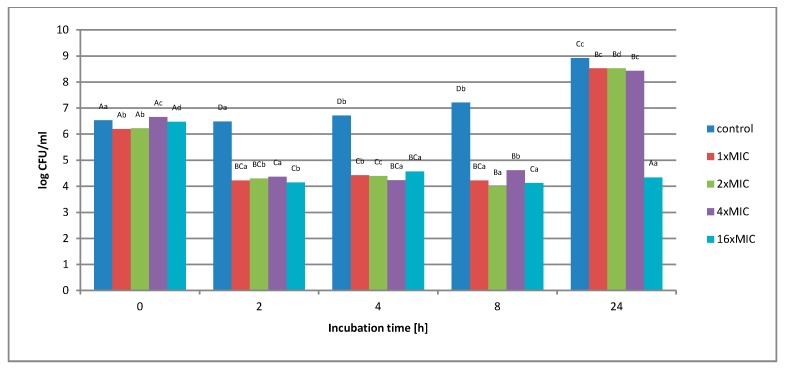
The effect of the aqueous extract at the concentration of 1 *×* MIC, 2 *×* MIC, 4 *×* MIC, and 16 *×* MIC on the survival of *Salmonella* Enteritidis in a matrix containing 3% meat extract during 24 h of incubation. A, B, C, D—the effect of matrix type on the change in the number of bacteria at the same time of storage; a, b, c, d—the effect of incubation time on changing the number of bacteria.

Under the influence of the concentration of 1 × MIC extract (16 mg*/*mL), a decrease in the number of bacterial cells from 1.6 *×* 10^6^ to 1.7 × 10^4^ colony-forming unit CFU*/*mL was observed in the first 8 h of incubation. After this period, an increase in the number from 1.7 × 10^4^ to 3.5 × 10^8^ CFU*/*mL was observed. The use of higher extract concentration, i.e., 2 × MIC (32 mg*/*mL), 4 × MIC (64 mg*/*mL), and 16 × MIC (256 mg*/*mL), caused the inhibition of bacterial growth by 2 log after 8 h of incubation, and this value was maintained until the end of the experiment. At none of the concentrations used in the control matrix was a complete inhibition of *Salmonella* Enteritidis growth was noted. Figure 5 shows the survival of *Salmonella* Enteritidis cells in an experimental matrix containing 3% meat extract in its composition.

The study found that the use of the extract at a concentration of 1 *×* MIC, 2 *×* MIC, and 4 *×* MIC did not inhibit the growth of the test bacterium after 24 h of incubation. Initially, a decrease in cell number was observed. The lower number of cells remained up to 8 h, while after this time an increase in the number of cells by about 4 log was observed. In turn, the use of an extract with a concentration of 16 *×* MIC (256 mg*/*mL) gradually reduced the number of *S.* Enteritidis cells in just 2 h of incubation from 3.0 *×* 10^6^ CFU*/*mL to 1.4 *×* 10^4^ CFU*/*mL. The value obtained after 2 h remained at a similar level until the end of the 24-h incubation.

**Figure 6:**
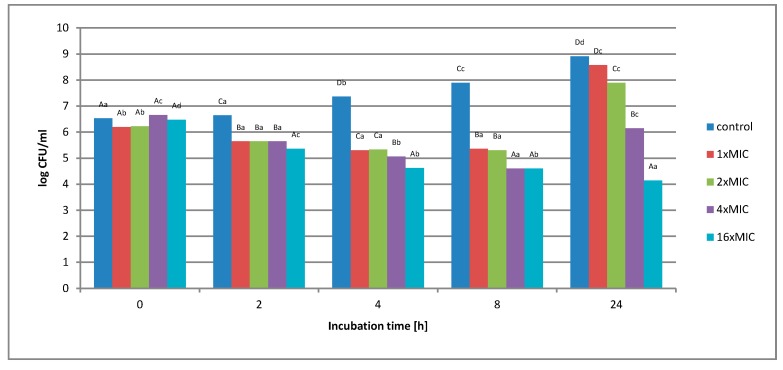
The effect of the aqueous extract at the concentration of 1 × MIC, 2 × MIC, 4 × MIC, and 16 × MIC on the survival of *Salmonella* Enteritidis in a matrix containing 6% meat extract during 24 h of incubation. A, B, C, D—the effect of matrix type on the change in the number of bacteria at the same time of storage; a, b, c, d—the effect of incubation time on changing the number of bacteria.

In the study of the experimental matrix containing 6% of meat extract (Figure 6), it was found that the use of an aqueous extract at a concentration of 1 *×* MIC and 2 *×* MIC did not inhibit the growth of the test bacterium. The highest concentration of 16 *×* MIC aqueous extract (256 mg*/*mL) gradually reduced the number of *S.* Enteritidis cells in the first hours of incubation. After 24 h, the number of CFU decreased from the level of 3.0 *×* 10^6^ CFU*/*mL to the level of 1.4 *×* 10^4^ CFU*/*mL.

**Figure 7:**
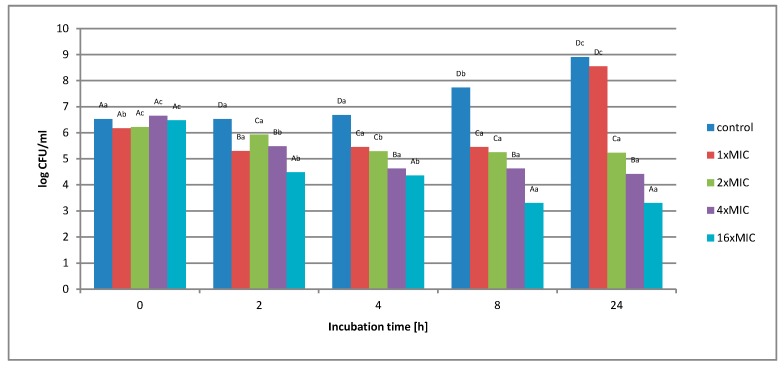
The effect of the aqueous extract at the concentration of 1 × MIC, 2 × MIC, 4 × MIC, and 16 × MIC on the survival of *Salmonella* Enteritidis in a matrix containing 12% meat extract during 24 h of incubation. A, B, C, D—the effect of matrix type on the change in the number of bacteria at the same time of storage; a, b, c, d—the effect of incubation time on changing the number of bacteria.

In the study of the experimental matrix containing 12% of meat extract (Figure 7), it was found that the use of an aqueous extract at a concentration of 1 × MIC did not inhibit the growth of the test bacterium. During the incubation, the number of bacteria significantly decreased after the first 2 h of incubation. However, in further measuring time points (4 and 8 h) a gradual growth of microorganisms was noted. After 24 h, the number of cells increased from 1.5 × 10^6^ CFU/mL to 3.6 × 10^8^. When using higher concentrations of the extract, i.e., 2 × MIC (32 mg/mL) and 4 × MIC (64 mg/mL), a slight decrease in the number of cells was noted over time. At 2 × MIC, the CFU was reduced by 1 log compared to the starting value. In contrast, the 4 × MIC concentration reduced the number of bacterial cells from 4.5 × 10^6^ to 2.6 × 10^4^ CFU/mL.

The highest concentration of 16 × MIC aqueous extract (256 mg/mL) significantly reduced the number of *S.* Enteritidis cells after just 2 h of incubation. After 24 h of incubation, the number of bacterial cells decreased from 3.0 × 10^6^ CFU/mL to 2 × 10^3^ CFU/mL, i.e., by 3.1 log.

##### Study of the Survival of *Listeria innocua* in Model Fluids Imitating Protein Food

**Figure 8:**
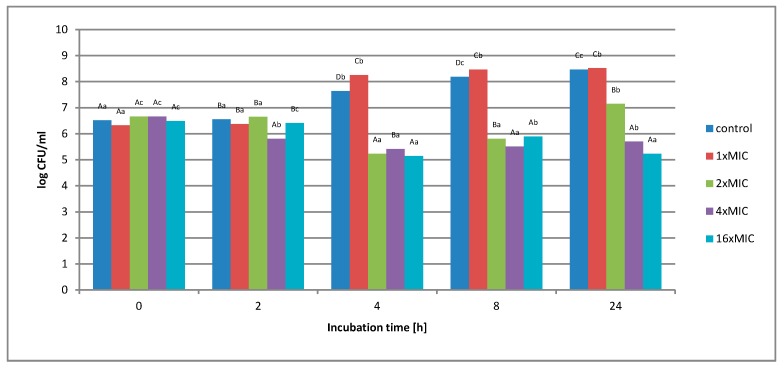
The effect of the aqueous extract at the concentration of 1 × MIC, 2 × MIC, 4 × MIC, and 16 × MIC on the survival of *Listeria innocua* in the control matrix during 24 h of incubation. A, B, C, D—the effect of matrix type on the change in the number of bacteria at the same time of storage; a, b, c, d—the effect of incubation time on changing the number of bacteria.

Figure 8 shows the effect of an aqueous extract on the survival of Gram-positive *Listeria innocua* in a control matrix, i.e., in TSB medium.

**Figure 9:**
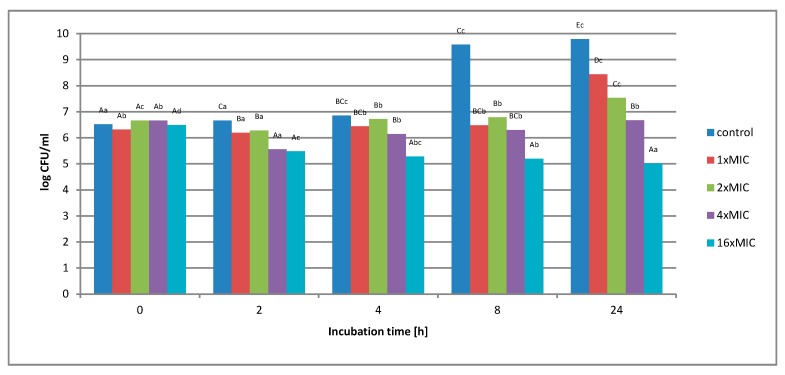
Effect of aqueous extract at a concentration of 1 × MIC, 2 × MIC, 4 × MIC, and 16 × MIC on the survival of *Listeria innocua* in a matrix containing 3% meat extract during 24 h of incubation. A, B, C, D—the effect of matrix type on the change in the number of bacteria at the same time of storage; a, b, c, d—the effect of incubation time on changing the number of bacteria.

Regardless of the concentration of the extract used, no complete inhibition of microbial growth was observed. Under the influence of 1 × MIC (16 mg/mL), an increase in the number of bacteria from 2.1 × 10^6^ to 3.3 × 10^8^ CFU/mL was observed. The use of twice the concentration of the extract (2 × MIC) caused an initial decrease in the number of bacterial cells. The lowest value was recorded in 4 h of measurement. After this time, however, cell proliferation and growth were noted. After 24 h of cultivation, the number of microorganisms increased from the initial value of 4.6 × 10^6^ to 1.4 × 10^7^ CFU/mL. At 4 × MIC extract concentration (32 mg/mL), a reduction in cell number was observed in the early hours of breeding. After 2 h, the number of cells dropped from 4.6 × 10^6^ to 6.4 × 10^5^, and this value was maintained until the end of the incubation. The highest concentration of 16 × MIC extract (256 mg/mL) caused the inhibition of bacterial growth by 1.4 log after 4 h of measurement, this value was maintained until the end of the incubation. Figure 9 shows the survival of *Listeria innocua* cells in the experimental matrix containing 3% of meat extract. The study found that the use of an aqueous extract at a concentration of 1 × MIC and 2 × MIC did not inhibit the growth of the test bacterium.

**Figure 10:**
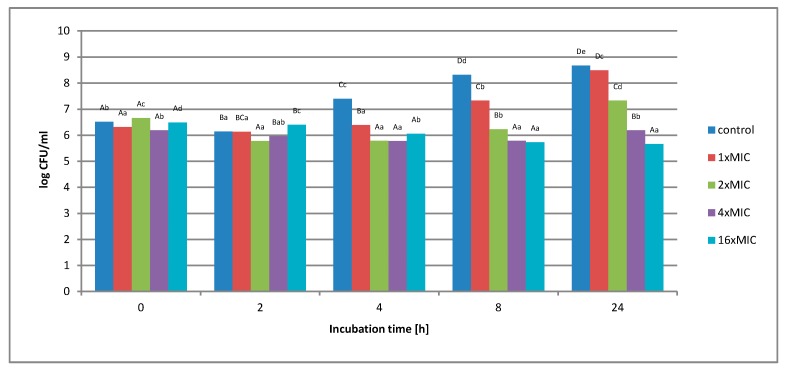
Effect of aqueous extract at a concentration of 1 × MIC, 2 × MIC, 4 × MIC, and 16 × MIC on the survival of *Listeria innocua* in a matrix containing 6% meat extract during 24 h of incubation. A, B, C, D—the effect of matrix type on the change in the number of bacteria at the same time of storage; a, b, c, d—the effect of incubation time on changing the number of bacteria.

After 24 h, 2 × MIC increased from 4.6 × 10^6^ CFU/mL to 3.4 x10^7^ CFU/mL, and, In turn, it maintained a similar number of cells for 64 mg/mL (4 × MIC), as for the zero hour, after 24 h of incubation (4.6 × 10^6^ to 4.7 × 10^6^). The highest concentration of 16 × MIC aqueous extract (256 mg/mL) gradually reduced the number of *Listeria innocua* cells in just 2 h of incubation. With the duration of the incubation, this value gradually changed. After 24 h, the number of cells decreased from 3.1 × 10^6^ CFU/mL to 1.1 × 10^5^ CFU/mL. However, even at the highest concentration of the extract, no bactericidal effect was observed during 24 h incubation. Figure 10 shows the survival of *Listeria innocua* cells in the experimental matrix containing 6% of meat extract.

The study found that the use of an aqueous extract at a concentration of 1 × MIC and 2 × MIC did not inhibit the growth of the test bacterium. After 24 h, the number of cells for 2 × MIC increased from the initial value of 4.6 × 10^6^ to 2.1 × 10^7^ CFU/mL. At the 4 × MIC extract concentration (64 mg/mL), the number of bacterial cells remained at the initial cell number after 24 h of incubation. The highest concentration of 16 × MIC aqueous extract (256 mg/mL) gradually reduced the number of *Listeria innocua* cells from 3.1 x10^6^ CFU/mL to 4.6 × 10^5^ CFU/mL.

**Figure 11:**
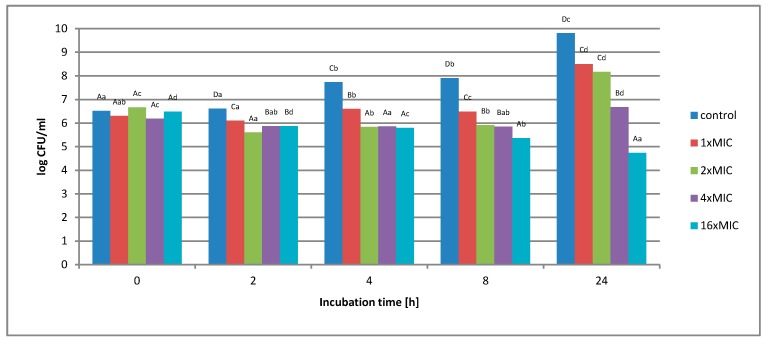
Effect of aqueous extract at a concentration of 1 × MIC, 2 × MIC, 4 × MIC, and 16 × MIC on survival of *Listeria innocua* in a matrix containing 12% meat extract during 24 h incubation. A, B, C, D—the effect of matrix type on the change in the number of bacteria at the same time of storage; a, b, c, d—the effect of incubation time on changing the number of bacteria.

No complete inhibition of bacterial growth was found at any of the concentrations used (Figure 11). At 1 × MIC extract concentration (16 mg/mL), an increase in the number of cells from the initial value of 2.0 × 10^6^ to 3.2 × 10^8^ CFU/mL was noted. In the case of a twice the concentration (2 × MIC - 32 mg/mL), a decrease in the number of bacterial cells was noted after 2 h of incubation. With the duration of the incubation, this value increased slightly at the 8-h time point. After this time, microbial cells grew rapidly. After 24 h of incubation, the number of cells increased from 4.6 × 10^6^ to 1.5 × 10^8^ CFU/mL. At a concentration of 4 × MIC (64 mg/mL), no significant microbial growth was noted. The number of cells after 24 h of incubation remained at a similar level as the initial value. The highest concentration of 16 × MIC extract (256 mg/mL) resulted in a reduction in cell number from an initial value of 3.1 × 10^6^ to 5.5 × 10^4^ CFU/mL.

Comparing the activity of the aqueous extract against the tested bacterial strains in matrices imitating protein food, it can be seen that *S.* Enteritidis cells were found to be more sensitive compared to *L. innocua* cells. However, at none of the extract concentrations used in the tested models, a complete reduction in the number of microorganisms was observed. The largest reduction in cell number was noted in the protein model containing 12% meat extract, where, in the case of *Salmonella* Enteritidis, bacterial growth inhibition and a 3-log reduction of cell number were observed, whereas, in relation to *L. innocua*, a 2-log reduction was observed. In the 3% and 6% meat extract models, a 2-log reduction in the number of *S.* Enteritidis cells was observed. In turn, relative to *L. innocua* in the model with a content of 3% and 6% meat extract observed only a 1-log reduction in cell number. There are no similar results in the literature regarding the study of rose fruit extracts for bacterial survival, and therefore this study findings were compared with the results of other authors who conducted similar studies for other plant substances.

Diao et al. [35] conducted research on the effect of various concentrations of essential oil on one of the food-borne bacteria. The subject of the analysis was *Shigella dysenteriae*, Gram-negative bacterium grown on PCA medium. The research was carried out for 24 h. According to the authors, the value of MIC −0.125 mg/cm^3^ caused a decrease in the number of cells able to survive during 12 h of incubation. The number of cells decreased by 11.67%. However, no complete inhibition of growth of the studied pathogen was noted, only its reduction. In turn, the use of a twice higher concentration of oil resulted in the complete inhibition of *Shigella dysenteriae* growth after 24 h of cultivation. The use of a higher concentration significantly reduced the number of bacteria. In another study, authors tested the effects of natural plant substances found in blueberry and blackberry extracts on bacterial survival. Extracts obtained from these plants are rich in phenolic compounds. The studies were conducted on two food pathogens (*Salmonella* Enteritidis and *Listeria monocytogenes*) in tryptic-soybean broth. The extract concentration was 46.25 ppm for blackberry and 24 ppm for blueberry. The study was conducted for 24 h. *Salmonella* was found to be relatively more sensitive to the extracts compared to *Listeria monocytogenes*. The growth of Gram-negative *Salmonella* was significantly inhibited at all measuring points. The study confirmed that the extracts can affect the growth of the tested strains. It has also been found that phenolic compounds are responsible for limiting the growth of the studied pathogens [36].

In another work, researchers analyzed the activity of ethyl acetate extract from Urtica dioica to *Bacillus subtilis*. The experiment was conducted in concentrations, i.e., 4.16 mg/mL, 8.33 mg/mL, and 16.67 mg/mL, at the initial density of bacterial suspension 105 CFU/mL during 48 h. The number of *Bacillus subtilis* cells decreased by 1 log at 1 × MIC and 2 × MIC extract concentrations after 16 h of incubation. Bacterial cultures were monitored up to 48 h and no bacterial re-growth was observed. The presented research results prove the possibility of using the extract to inhibit or delay the growth of pathogens [37].

Muniandy et al. [38] studied the effect of oregano extract on the survival of *E.coli*, *K. pneumonia*, *P. aeruginosa*, *S. aureus*, and *P. mirabilis*. The initial density of the bacterial suspension was 5 × 10^5^ CFU/mL for each bacterium, while the concentration of inhibitory substance used in the study was 0.5 × MIC, 1 × MIC, and 2 × MIC. The number of viable cells was determined after 0, 3, 6, and 24 h. The mean reduction in the number of cells ranged from 0.60 to 3.69 log10 CFU/mL after 3 h and between 1.43 to 7.61 log10 CFU/mL after 6 h. In the case of 2 × MIC extract concentration, almost a complete inhibition of growth of tested microorganisms was noted as early as 6 h of incubation. On the other hand, when using 0.5 × MIC and 1 × MIC concentrations, no complete inhibition of microbial growth was found even after 24 h of incubation.

### 2.2. Antioxidant Activity of Extracts

According to previous reports, polyphenols and flavonoids in *Rosa rugosa* exhibit a variety of bioactivities, especially antioxidant properties [39,40]. Therefore, with different approaches and mechanisms, the four most common antioxidant activity assays, ferric reducing antioxidant power (FRAP), 2,2-diphenyl-1-picrylhydrazyl (DPPH), 2,2’-azinobis(3-ethylbenzothiazoline-6-sulfonic acid (ABTS), and oxygen radical absorbance capacity (ORAC), were carried out in vitro. The results obtained when testing the antioxidant properties of rose fruits extracts using four different tests are shown in Table 1. The ethanolic extracts from rose fruits showed higher antioxidant activity compared to other tested extracts. The antioxidant activity of the tested ethanolic extract (E), measured by the ORAC method, was very high and amounted to 6.8 mmol per gram. The results obtained by the ORAC method for aqueous extracts were about three times lower compared to ethanolic extracts.

The samples obtained by supercritical extraction showed much lower activity (1.7 µM Trolox/g – DPPH^•^; 14 µM Trolox/g – ORAC, 9.4 µM Trolox/g – FRAP, 1.7 µM Trolox/g – ABTS^• +^). The use of different extraction solvents results in different antioxidant compositions. The polyphenolic compounds present in rose fruits dissolve well in ethanol or in ethanol/water mixtures. Low values of antioxiadant properties of the supercritical extracts indicate that they do not contain as much polyphenols as ethanol extracts, despite the use of ethanol as a modifier. The yellow color of the supercritical extract may indicate the presence of carotenoids present in rose fruits, but their contribution to antioxidant activity is much lower than that of polyphenolic compounds. Machmudah et al. [41], in the supercritical rosehip (fruits with seeds) extract, also found the presence of carotenoids, including mainly lycopene and carotene, which has an orange color.

It is worth underlining the effect of different solvents on antioxidant activity. In this study, ethanolic extracts had markedly higher antioxidant capacities than those of aqueous extracts. Therefore, ethanol is a more effective solvent for extraction of antioxidant compounds of Rosa species. This result is in agreement with the findings of Taneva et al. [42] and Franco et al. [43]. Ilbay et al. [44] found methanol extraction to be three-fold more effective than water extraction. Higher levels of phenols and antioxidant abilities were also found in the methanol and/or ethanol extracts of other plant species as compared to the water extracts of some authors [45,46].

## 3. Materials and Methods

### 3.1. Plant Material

Fresh fruits (including seeds) of *Rosa rugosa* were collected from plantation of the company “Polska Róża” located in Kotlina Kłodzka (16^o^39′E 50^o^27′N, Poland). Soil fertilization and shrub cutting were performed in accordance with the cultivation recommendations for this species. The fruits were collected in the first half of October 2017, at the stage of collective maturity. The temperature during the vegetative period was close to the annual averages. There were no extremes that could affect normal development of *Rosa rugosa*. The seedlings of *Rosa rugosa* were planted into a loose loam type soil mixture. The soil was slightly moist with added manure. The pH of the soil was about 6–6.5. Before planting and during the first year after the planting, no mineral fertilizers were used. In early spring, plants were trimmed 20 cm from root collar. Compound fertilizers were used during following years of plants growth. Fertilization was performed until the end of April. A longer fertilization period would have caused a longer vegetation period, which in turn would result in worse adaptation to the winter conditions. The raw material was stored until the beginning of the study at −18 °C. Before starting the research work, the rose fruits were subjected to pretreatment, which included washing under running water and manual dressing so as to separate damaged, overripe, and rotten fruits, unsuitable for further processing.

### 3.2. Chemicals

2,2′-azinobis-(3-ethylbenzothiazoline-6-sulfonic acid) (ABTS), 6-hydroxy-2,5,7,8-tetramethyl-chroman-2-carboxylic acid (Trolox), (2,4,6-tris (2-pyridyl)-1,3,5-triazine) (TPTZ) fluorescein (3‘,6‘-dihydroxyspiro[isobenzofuran-1[3H],9‘[9H]-xanthen]-3-one), 2,2-diphenyl-1-picrylhydrazyl (DPPH), were provided by Sigma–Aldrich Chemical Co. (St. Louis, MO, USA). Hydrochloric acid, ferrous sulfate, ferric chloride, methanol, and ethanol were provided by POCH (Gliwice, Poland). All the chemicals used were of analytical grade.

Spectrophotometric determinations were made using a spectrophotometer UV-VIS Evolution 60S (Thermo Scientific, USA). ORAC was performed in a Hitachi F-7000 spectrofluorimeter with an adapter for measurements on microplates (Hitachi High-Tech, Tokyo, Japan).

### 3.3. Preparation of Extracts

#### 3.3.1. Aqueous Extract (A)

Periodic single-stage extraction of the raw material was carried out using distilled water with a 1:2 ratio of raw material to solvent. Extractions were carried out using a semi-technical prototype device for extraction and distillation of herbs for 2 h at a temperature of 60 °C ± 5 °C. Next, the extracts were concentrated in a rotary evaporator (Rotavapor R-205, Büchi, New Castle, DE, USA). The following temperatures were used: bath heating 60 °C, condensate 40 °C, cooling water 20 °C. The concentrated extract was frozen to −80 °C and then lyophilized for 72 h (Alpha 1-4, Christ, Osterode am Harz, Germany).

#### 3.3.2. Ethanolic Extract (E-, E1)

The procedure for obtaining ethanol extracts was identical to the aqueous extract. The only difference was the solvent used, which was a 40% or 60% ethyl alcohol solution prepared from distilled water, 96% ethyl alcohol (POCH, Gliwice, Poland).

#### 3.3.3. Supercritical Extract (S)

Extraction of rose fruits with carbon dioxide in a supercritical state was carried out on a semi-automatic, multi-station Supercritical fluid extraction (SFE) system (MV-10 ASFE System, Waters, and Milford, MA, USA). The process was automatically controlled by a computer using Chromo ScopeTM software. As the modifier, 96% ethanol was used. Extractions were carried out in the following conditions: temperature—60 °C; pressure—280 bar; time—30 min (static time); 10 min (dynamic time); CO_2_ flow—4 mL/min; and modifier flow—1 mL/min. Next, the extracts were concentrated in a rotary evaporator (Rotavapor R-205, Büchi, New Castle, DE, USA). The following temperatures were used: bath heating 60 °C, condensate 40 °C, cooling water 20 °C. The concentrated extract was frozen to −80 °C and then lyophilized for 72 h (Alpha 1-4, Christ, Osterode am Harz, Germany).

#### 3.3.4. Enzymatic Extract (P)

To prepare the extract, 1 kg of rose fruits and 2 kg of distilled water, which had previously been acidified with concentrated hydrochloric acid to pH = 2, and pepsin to obtain a concentration of about 1.0%, were used. The samples were then incubated at 37 °C for 48 h. Hydrolysis was stopped by cooking for 10 min. Next, the extracts were concentrated in a rotary evaporator (Rotavapor R-205, Büchi, New Castle, DE, USA) in order solvent evaporation. The following temperatures were used: bath heating 60 °C, condensate 40 °C, cooling water 20 °C. The concentrated extract was frozen to −80 °C and then lyophilized for 72 h (Alpha 1-4, Christ, Osterode am Harz, Germany).

**Figure A1:**
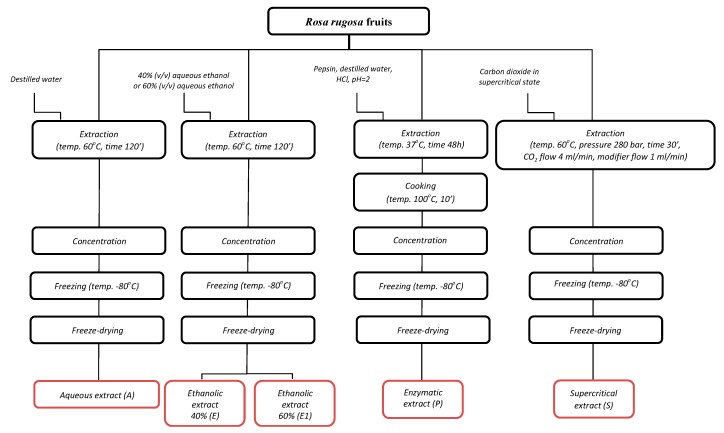
The scheme for preparing extracts from *Rosa rugosa* fruits.

The scheme for preparing extracts is shown Appendix (Figure A1).

All extracts were prepared in triplicate and the obtained samples were marked with the following symbols: A—aqueous extract, E—ethanolic extract (40% *v/v* aqueous ethanol), E1—ethanolic extract (60% *v/v* aqueous ethanol), S—supercritical carbon dioxide extract, P—enzymatic extract.

### 3.4. Antibacterial Activity

#### 3.4.1. Test Microorganisms and Preparation of Inoculum

Reference strains originated from the American Type Culture Collection (ATCC, Manassas, VA, USA), and the strain isolated from food originated from the Department of Biotechnology, Microbiology and Food Evaluation (SGGW, Poland). The study of aqueous extract (A) and ethanolic extract (E) used 5 strains of Gram-positive bacteria (*B. cereus* ATCC 11778, *E. faecalis* ATCC 29212, *S. aureus* ATCC 25923, *S. epidermidis* ATCC 12228, *L. innocua* SGGW) and 5 strains of Gram-negative bacteria (*E. coli* ATCC 25922, *K. pneumoniae* ATCC13883, *P. mirabilis* ATCC 35659, *P. aeruginosa* ATCC 27853 *S.* Enteritidis ATCC 13076). The study of other extracts (E1, S, P) used only 1 strain of Gram-positive bacteria (*S. aureus* ATCC 25932) and 1 strain of Gram-negative bacteria (*S*. Enteritidis ATCC 13076). The bacterial strains were cultured on Mueller-Hinton Agar (BTL, Poland) and incubated at 37 °C for 24 h. The bacterial inocula were prepared in sterile 0.85% NaCl (*w*/*v*) solution to reach a population of approximately 10^8^CFU × mL^−1^.

#### 3.4.2. The Minimum Inhibitory Concentration (MIC) and Minimum Bactericidal Concentration (MBC)

Minimum inhibitory concentration (MIC) and minimum bactericidal concentration (MBC) of the extracts were determined using the method of serial microdilutions [47]. To this end, double series of 10 tubes containing 2 mL of sterile Mueller-Hinton Broth (BTL, Poland) were prepared. In total, 2 mL of the test extract was added to the first tube from each series, thus obtaining 4 mL of solution in the first tube. The contents of the tube were mixed thoroughly and then transferred 2 mL of the solution to the next tube in the series and mixed again. The same procedure was followed until the end of the series. For the last tube, 2 mL was discarded to equalize the volume of the remaining tubes in series. Bacterial inoculum was added to the prepared ranks and the control sample containing only liquid medium 0.1 mL, obtaining in each tube from the series a concentration of about 5 × 10^5^ CFU/mL. Series incubations were carried out at 37 °C for 24 h. The MIC value was defined as the lowest concentration of extract, in which no visual growth of bacteria was noted.

MBC examination involved the transfer of 0.1 mL bacteria culture from each well where no growth was observed on the plates with Mueller-Hinton Agar (BTL, Poland). The plates were incubated at 37 °C for 24 h. The growth of colonies on the plates was verified after that incubation time. MBC was defined as the lowest concentration of extract, which resulted in a complete reduction of bacteria.

#### 3.4.3. Preparation of Model Fluids Imitating Protein Food

As food imitation models, the following were used: control medium, TSB broth, and experimental medium, i.e., TSB broth with the addition of meat extract in a concentration of 3%, 6%, and 12%. The inhibiting factor was the aqueous extract, while the biological material was a Gram-negative and Gram-positive bacterial strain. The aqueous extract was used because of the stronger antimicrobial effect on bacteria, as well as for economic reasons. *Salmonella* Enteritidis was selected from Gram-negative bacteria, while *Listeria innocua* was selected from the group of Gram-positive bacteria. Both bacteria used in the study were selected because of the widespread occurrence in food and the high risk for humans.

**Figure A2:**
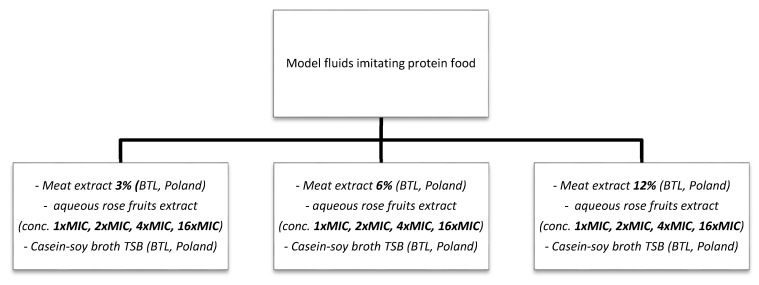
The scheme for preparing model fluids imitating protein food.

The scheme for preparing model fluids imitating protein food is shown Appendix (Figure A2).

#### 3.4.4. Survival of Selected Bacterial Strains Using the Time-Kill Method

The time-kill method of synergy testing was performed by the broth macrodilution technique and followed the guidelines set by the National Committee for Clinical Laboratory Standards [48]. The survival of microorganisms by the time-kill method was assessed by exposing the suspension of the selected bacterial strain to selected concentrations of the aqueous extract and observing the survival of the cells at selected time intervals. The following concentrations of the aqueous extract 1 × MIC (16 mg/mL), 2 × MIC (32 mg/mL), 4 × MIC (64 mg/mL), 16 × MIC (256 mg/mL) were tested.

The analysis was carried out on a control medium, i.e., in TSB broth and in food simulant, i.e., TSB broth with 3%, 6%, and 12% meat extract. In each test tube there was a total of 3 mL of the mixture: with a suitable medium, inhibitory substance and bacterial suspension with an initial density of 10^6^ CFU/mL. The cultures were incubated for 24 h at 37 °C, and during the cultivation samples of 0.1 mL volume were taken at 0, 2, 4, 8, and 24 h time points. The samples were then diluted appropriately in physiological saline. Appropriate dilutions of 1 mL were taken transferred to Petri dishes with TSB medium. The plates were incubated at 37 °C for 24 h. After this period, the colonies were counted.

The number of microorganisms (L) in 1 mL of model fluids imitating protein food was calculated from the following formula:

L = C/[(N_1_ + 0.1 N_2_) ∗ D] (1)

where C is the sum of colonies grown on all plates (between 15–300);

D is the lowest calculated dilution;

N_1_ is the number of plates from the first calculated dilution; and

N_2_ is the number of platelets from the second dilution count.

The reduction in the number of microorganisms was determined in log_10_ degrees per unit of time.

### 3.5. Antioxidant Activity

In order to measure the antioxidant activity, four methods were used with different mechanisms of action that allowed for a complete assessment of the antioxidant activity of the tested extracts. (1) DPPH Free Radical Scavenging Assay. The DPPH (2,2-diphenyl-1-picrylhydrazyl) free radical scavenging activity was determined using the method proposed by Sanna et al. [49]. (2) ABTS Radical Cation Decolorization Assay. The ABTS (2,2′-azinobis (3-ethylbenzothiazoline-6-sulphonic acid) radical cation decolorization assay was applied according to the methodology described by Re et al. [50]. (3) The Ferric Reducing Antioxidant Power (FRAP) analysis was performed according to the procedure described by Benzie and Strain [51]. (4) The ORAC (Oxygen-Radical Absorbance Capacity) was performed according to Ou et al. [52]. All results are expressed in Trolox equivalents—μmol of TE/g of freeze-dried extracts.

### 3.6. Statistical Analysis

The microbiological population results were log transformed for statistical analysis. All results are expressed as the mean ± standard deviation (SD) of three replicates. Statistical analysis was conducted using Statistica version 10.0 (StatSoft Poland, Cracow, Poland). Significant differences (*p* < 0.05) between average responses were evaluated with the use of one-way ANOVA with Tukey test.

## 4. Conclusions

Due to the increasing resistance of bacteria, as well as the tendency to reduce the use of chemical additives, there is a need for new antibacterial and antioxidant agents. Undoubtedly, the alternative is the world of plants and extracts derived from them, which, due to their natural origin, are gaining recognition among consumers. We have confirmed that the traditional extraction and freeze-drying of rose fruits is still suitable for the food industry due to obtaining extracts with good antibacterial and antioxidant properties and the use of bio-solvents, such as water or ethanol, which are easily available in high purity and completely biodegradable. The obtained lyophilized aqueous, alcohol, and enzymatic extracts were characterized by high antioxidant activity, determined in four tests FRAP, DPPH^•^, ORAC, and ABTS^•+^ tests. The rose fruits aqueous extract showed the highest inhibitory activity against most of the 10 bacterial strains tested. The reduction in the number of bacterial cells in matrices imitating protein food depended on the concentration of the extract used. The obtained test results confirm the possibility of using rose extracts to extend the microbiological stability of food. Due to the high availability of the raw materials and the relatively simple procedure for obtaining them, extracts of rose fruits (*Rosa rugosa*) can be considered an interesting addition to diet, food, or dietary supplements.

## Figures and Tables

**Table 1:** Antiradical activity of freeze-dried extracts.

Sample	DPPH ^●^(µmol TE/g for Trolox Equivalents/g Freeze-Dried Extract)	ORAC (µmol TE/g for Trolox Equivalents/g Freeze-Dried Extract)	FRAP (µmol TE/g for Trolox Equivalents/g Freeze-Dried Extract)	ABTS^●+^ (µmol TE/g for Trolox Equivalents/g Freeze-Dried Extract)
Aqueous extract (A)	887.7 ± 39.0 b	2142 ± 69.1 b	880.6 ± 3.5 c	567.0 ± 3.2 c
Ethanolic extract (E)	1069.7 ± 2.7 d	6801 ± 38.1 d	1016.9 ± 1.2 e	605.0 ± 1.6 e
Ethanolic extract (E 1)	994.6 ± 32.2 c	5019 ± 26.7 c	980.1 ± 5.9 d	574.8 ± 8.9 d
Supercritical extract (S)	1.7 ± 0.1 a	14 ± 0.3 a	9.4 ± 0.3 a	1.7 ± 0.1 a
Enzymatic extract (P)	865.8 ± 13.7 b	2098 ± 16.5 b	865.3 ± 4.5 b	560.3 ± 1.3 b

Different letters in the same column indicate significantly different (*p* < 0.05).
